# Synthesis and Characterization of Ciprofloxacin Loaded Star-Shaped Polycaprolactone–Polyethylene Glycol Hydrogels for Oral Delivery

**DOI:** 10.3390/mi14071382

**Published:** 2023-07-06

**Authors:** Wan Khartini Wan Abdul Khodir, Mohamad Wafiuddin Ismail, Shafida Abd Hamid, Rusli Daik, Deny Susanti, Muhammad Taher, Vincenzo Guarino

**Affiliations:** 1 Department of Chemistry, Kulliyyah of Science, International Islamic University Malaysia Kuantan Campus, Bandar Indera Mahkota, Kuantan 25200, Pahang, Malaysia; 2 Synthetic and Functional Materials Research Group (SYNTOF), Kulliyyah of Science, International Islamic University Malaysia Kuantan Campus, Bandar Indera Mahkota, Kuantan 25200, Pahang, Malaysia; 3 Department of Chemical Sciences, Faculty of Science & Technology, Universiti Kebangsaan Malaysia, Bangi 43600, Selangor, Malaysia; 4 Department of Pharmaceutical Technology, Kulliyyah of Pharmacy, International Islamic University Malaysia Kuantan Campus, Bandar Indera Mahkota, Kuantan 25200, Pahang, Malaysia; 5 Institute of Polymers, Composites and Biomaterials, National Research Council of Italy, Mostra d’Oltremare Pad.20, V.le J.F.Kennedy 54, 80125 Naples, Italy

**Keywords:** PCL arms, star co-polymers, hydrogels, ciprofloxacin, drug delivery

## Abstract

The administration of poorly water-soluble drugs represents a relevant problem due to the low body fluids transport efficiency through hydrophilic hydrogels. Star-shaped co-polymers, i.e., amphiphilic polymers such as those with a hydrophobic core and a hydrophilic outer shell, can be used to improve weak interactions with drugs, with relevant benefits in terms of administration and controlled delivery. In this work, two different co-polymers, four-arm star-shaped PCL–PEG and six-arm star-shaped PCL–PEG, were synthesized via ring-opening polymerization to be loaded with ciprofloxacin. ^1^H-NMR and FTIR analyses confirmed that PCL arms were successfully grafted to the mPEG backbone, while DSC analysis indicated similar crystallinity and melting point, ranging from 56 to 60 °C, independent of the different co-polymer architecture. Therefore, both star-shaped PCL-PEGs were investigated as cargo device for ciprofloxacin. No significant differences were observed in terms of drug entrapment efficiency (>95%) and drug release, characterized by a pronounced burst followed by a slow sustained release, only slightly affected by the co-polymer architecture. This result was also confirmed with curve fitting via the Korsmeyer–Peppas model. Lastly, good antibacterial properties and biocompatibility exhibited in both star-shaped PCL–PEG co-polymers suggest a promising use for oral delivery applications.

## 1. Introduction

The development of effective medicinal products from poorly water-soluble drugs continues to be a challenging task with the current pharmaceutical technology; however, this is crucial for the treatment of a wide range of diseases. Approximately 40% of drugs currently approved for market use and almost 90% of molecules still in the discovery phase suffer from poor water solubility, which ultimately results in limited absorption and low bioavailability when orally administered [1,2,3,4,5,6,7]. Various approaches and preparations have been tested to overcome this limitation. Amphiphilic star-shaped polymers have more stable structures than amphiphilic linear co-polymers due to their covalently branching point on the core. Their architecture, consisting of hydrophobic and hydrophilic polymers, provides useful properties such as the protection of the drug from premature degradation and the elimination of additional surfactants in drug formulations [8,9,10,11]. Many studies have reported that complex star architectures can potentially act as cargo to deliver pharmaceutical drugs and biologicals (peptides, nucleic acids) in a targeted area to treat/manage pain in the presence of bacterial infection or cancer activity [12,13,14,15]. Hence, the idea of properly designing the co-polymer architecture to obtain a controlled drug-release pattern is growing [16,17].

In this context, polyesters/polyether-based amphiphilic star-shaped polymers, such as poly-caprolactone-b-poly(ethylene glycol) (PCL–PEG), enable the increase in the encapsulation of a hydrophobic drug via a hydrophobic–hydrophobic interaction or the conjugation of a drug with PCL arms [18,19,20]. The co-polymerization of PCL with other lactones or glycolides/lactides can influence the hydrolysis mechanism of PCL, promoting water intake and, consequently, increasing the hydrolysis rate of the PCL blocks [21]. In addition, PEG, working as a hydrophilic part, increases the solubility of the cargo [22,23], also allowing a controlled release with a global reduction in the uptake of harmful immunoglobins [24,25]. In recent years, many researchers have focused on the synthesis of star-polymer-based hydrogels as an attractive approach for the development of more efficient dosage forms [26] based on co-polymers with selected features in terms of water absorption, swelling, and degradation [27,28,29]. 

In this study, amphiphilic star shapes of polycaprolactone–polyethylene glycols consisting of four and six arms were synthesized. In detail, the hydrophobic, slowly degradable arms of PCL were grafted to hydrophilic PEG segments to form a star-shaped architecture. Ciprofloxacin (Cipro) was used as a model poorly water-soluble drug to validate the use of star-shaped PCL–PEG as a carrier for the oral administration of hydrophobic drugs. 

## 2. Materials and Methods

### 2.1. Materials

ε-Caprolactone, succinic anhydride, pentaerythritol, di-pentaerythritol, 4-(dimethylamino)pyridine (DMAP), 1,3-dicyclohexylcarbodiimide (DCC), ciprofloxacin 99%, and carbomer 940 were purchased from Across Organic (Geel, Belgium) and were used without further purification. Monomethoxy poly(ethylene glycol), (mPEG) with Mn = 5.0 kDa, and Tin(2-ethylhexanoate)2 (Sn(Oct)2) were purchased from Sigma-Aldrich (St. Louis, MO, USA). Methyl paraben, ethyl paraben, and trifluoroethanol (TFE) were purchased from Merck. Pvt. Ltd. (Darmstadt, Germany), and trieathanolamine (TEA) was purchased from Q-rec (Jaipur, India). All chemicals used in this study were of analytical grade and used without further purification. Bacterial strains of *P. aeruginosa* (ATCC 27853), *E. coli* (ATCC25922), *E. faecalis* (ATCC29212), and *S. pyogenes* (ATCC19615) were procured from the American Type Culture Collection (ATCC), Manassas, VA, USA.

### 2.2. Synthesis and Optimization of Star Polymers PCL-PEG

**Figure 1:**
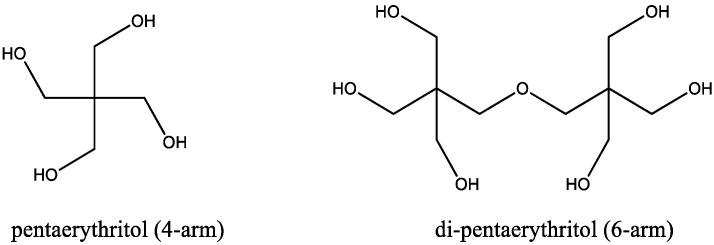
Initiator for four-arm star-shaped polymer (pentaerythritol) and six-arm star-shaped polymer (di-pentaerythritol).

In the preparation of star polymer, two types of initiators were used. Pentaerythrytol was used to develop four-arm star polymer, and di-pentaerythritol for six-arm star polymer (Figure 1). The molar ratio of the ɛ-caprolactone is based on the number of hydroxyl groups of the initiator to obtain an analogous star-shaped PCL. 

For the four-arm star-shaped PCL (4Star-PCL) and six-arm star-shaped PCL (6Star-PCL), approximately 10 g (0.087 mol) of ɛ-caprolactone was mixed with the pentaerythritol/di-pentaerythritol (0.27 mmol) in a 100 mL round bottom flask. Then, the mixture was heated to 110 °C, and upon homogeneity, 0.005 g (0.1 mmol) stannous octoate catalyst was added. The flask was purged with nitrogen and covered with parafilm. All reactions were maintained using a silicone oil bath at the specified reaction temperature of 110 °C for approximately 24 h. The polymers were isolated via precipitation into cold diethyl ether, followed by several washes with copious amounts of the precipitating solvent, and dried under vacuum for 48 h. 

**Figure 2:**
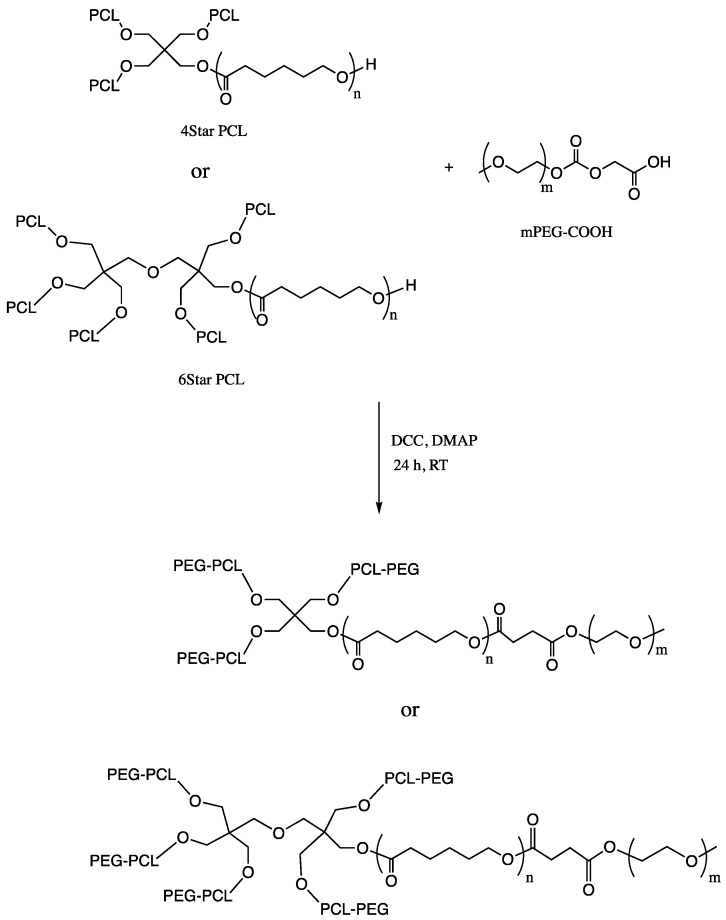
Synthesis of star polymers of PCL-PEG.

PCL-PEG star polymer was then synthesized by reacting carboxylated mPEG, 4Star-PCL, and 6Star-PCL (Figure 2). mPEG-COOH (0.1 mmol), DMAP (0.2 mmol), DCC (0.2 mmol), and star PCL (0.025 mmol) were dissolved in 10 mL of methylene chloride. The reaction was carried out at room temperature for 48 h under nitrogen. After the removal of dicylcohexylcarbodiurea via filtration, the polymer was precipitated via cold diethyl ether and dried at room temperature for 48 h. For the six-arm PCL-PEG, the same procedure was applied but using a different initiator (di-pentaerythritol).

### 2.3. Characterization of Star Polymers PCL and Star Polymers PCL-PEG

The functional groups of star polymers were identified using Perkin-Elmer Spectrum 400FTIR/FT-NIR spectrometer. Solid samples were ground with anhydrous potassium bromide (KBr). Infrared spectra were recorded in the region 4000–400 cm^−1^. 1H-NMR spectra of the polymers dissolved in deuterated chloroform (CDCl_3_) were recorded using a Varian 400 spectrometer at 400 MHz. In this case, Tetramethylsilane (TMS) was used as the internal reference, while all chemical shifts were reported in parts per million (ppm). Thermogravimetric TGA/DTG analysis was carried out using a Thermogravimetric Analyzer (Mettler Toledo, Columbus, OH, USA) on a crucible of aluminum containing 5 mg of sample. All the tests were performed by imposing a temperature range from 30 to 600 °C with a heating rate of 10 °C under nitrogen (N^2^) flow (50 mL/min). The differential scanning calorimetry (DSC) analysis was carried out using a Differential Scanning Calorimeter ((Mettler Toledo, Columbus, OH, USA) under nitrogen flow (10 mL/min) by imposing a heating rate of 10 °C/min into a range from 30 to 400 °C. The glass transition temperature (T_g_) and crystalline melting temperature (T_m_) were recorded.

### 2.4. Preparation of Ciprofloxacin-PCL-PEG Star Polymer-Based Hydrogel

4Star PCL-PEG and 6Star PCL-PEG (ca. 300 mg) were first diluted in TFE and then mixed with ciprofloxacin (90 mg) via magnetic stirring for 12 h until it formed a homogeneous mixture. Methyl paraben (15 mg) and propyl paraben (9 mg) were added in the formulation as preservative agent. Meanwhile, Carbopol resin was dispersed in 15 mL of deionized water for 12 h until it became a clear solution. Both mixtures were then mixed together and stirred for 24 h. The final mixture was neutralized with dropwise addition of trimethylamine until it formed a stable gel stabilized via storage for 24 h at room temperature. Standard formulation without ciprofloxacin loading was also prepared as control (STN6 and STN6).

### 2.5. Characterization of Hydrogels

For all the formulations, pH was measured using digital pH meter (Mettler Toledo, Columbus, OH, USA). The equipment was calibrated via a standard buffer solution at 4.0, 7.0, and 9.0. The pH probe was dipped into the solution for two minutes until a stable pH reading was obtained. pH measurements were performed in triplicate, and the average value was considered. A Brookfield digital viscometer (model DV-III, Middleboro, MA, USA) was used to measure the gel viscosity. After 30 minutes of rest, a spindle rotation (level 4) of 50 rpm was applied to the solution, and the viscosity measure was collected after 2 min.

### 2.6. Drug Entrapment Efficiency

The prepared gels (100 mg) were dissolved in 100 mL of phosphate-buffered solution. The flask containing gel solution was shaken for 30 min on mechanical shaker. Then, the sample was analyzed via UV spectrophotometer. The calibration curve (y = 0.1247x + 0.3078; R^2^ = 0.9979) was reported in Figure S1. The entrapment efficiency was calculated using the following equation [30]:

$$Percentage\;of\;Drug\;Entrapment=\frac{W-w}{W}\times 100$$

*W*: Amount of drug used in formulation
*w*: Amount of drug found in the solution

### 2.7. In Vitro Drug Release

The release rate of ciprofloxacin was determined using Dissolution Testing Apparatus basket method [31,32]. The hydrogel within a meshed basket was placed into a vessel containing 900 mL of phosphate-buffered solution at a pH equal to 7.4 ± 0.2. The vessel’s rotation speed was set at 100 rpm. At specific time intervals (1, 2, 3, 4, 5, 6, and 7 h), a 5 mL sample solution was collected and subsequently filtered through a membrane. The sample solution was collected hourly at different time intervals (1, 2, 3, 4, 5, and 6 h) and filtered. The same volume of fresh dissolution medium at the same temperature was added to replace the amount withdrawn after each sampling. The absorbance was measured at 270 nm using a UV/Vis spectrophotometer. Cumulative percentage of drug release was calculated in agreement with the standard curve.

The release kinetics of ciprofloxacin was investigated using four different models: zero-order model, first-order model, Hixson–Crowell, Higuchi, and Korsmeyer–Peppas model [33]. The model with highest linearity (or correlation coefficient, r^2^ value), i.e., zero-order model, will be considered for in vitro studies.

Zero-order release: Q_t_ = k_0_ t

where Q_t_ is the total drug release at time, t (in percentage concentration), and k_0_ is the constant of zero-order model (in concentration/time).

First-order release model: Log C = Log C_0_ − kt/2.303

where Q_0_ is the initial concentration of the drug, k is the constant of first-order model, and t is the time.

Higuchi model: Q_t_ = K_H_ t^1/2^

where Q is the amount of drug released in time, and t and KH are the Higuchi dissolution constants. The amount of drugs released reported as function of the square root of time, fitted a straight line.

Korsmeyer–Peppas release model: M_t_/M = k_kp_ t^n^

where M_t_ and M are the amount of drug at time t and loaded into the drug delivery system, respectively, and k is the kinetic constant related to the drug delivery system.

“n” is the release exponent that depends on the type of transport, geometry, and polydispersity of the solute and is strictly related to the solute transport mechanism: Fickian diffusion (n = 0.5), anomalous transport (0.5 < n < 1), and pseudo-Fickian diffusion (n < 0.5).

### 2.8. Antimicrobial Assay (Well Diffusion)

The bacteria were cultured on Mueller Hinton Agar (MHA) for 24 h at 37.5 °C. In vitro antibacterial activity in the gels was evaluated using agar well diffusion method [30]. A sterile cotton swab was used to spread the microbial inoculums onto the surface of the agar plates. In order to make wells (5 mm) on the inoculated agar plates, sterile pipette tips were used by punching holes in the agar. A total of 15 ± 1 mg of the formulations was weighed and placed into each well. Ciprofloxacin unloaded samples were used as a control for all the bacterial tests. The agar plates, incubated at 37 °C, were observed to detect the halo at 3, 6, 12, and 24 h of incubation. The inhibitory activity was measured in terms of average diameter of halo region. All tests were performed in triplicate.

### 2.9. Cell Culture and MTT Cell Assay

Human gingival fibroblasts, HGF-1 (ATCC^®^ CRL-2014^TM^—American Type Culture Collection—Manassaa, VA, USA), were used for biocompatibility studies. Cells were cultured in Dulbecco’s modified Eagle’s medium (DMEM) containing 10% fetal bovine serum.

4SF, 6SF, STN4, and STN6 samples were dissolved in 10 mL of DMEM medium (10 µg/mL). Then, the medium was filtered through a 0.22 mM filter paper and kept as a mother solution. Two dilutions were prepared to reach a final concentration of samples equal to 8 µg/mL and 5 µg/mL. HFG-1 (7 × 10^4^ cells/mL) was seeded into 96-well plates and incubated for 4 days until it reached the confluence. Then, culture medium was removed, washed with phosphate buffer, and replaced with complete (100 µL) medium containing 5, 8, and 10 µg/mL of gel solution for all four investigated types. Experiments were conducted in triplicate (n = 9).

The cytotoxicity tests were performed using Methylthiazolyldiphenyl-tetrazolium bromide (MTT) assay. The MTT solution, 10 µg/mL per well, was kept in incubator at 37 °C, 5% CO_2_ for 3 h. DMSO solution was then added to each well for 1 h to ensure complete solubilization of formazan crystals and the light absorbance at 570 nm wavelength, measured in triplicate via a microplate reader (Tecan Inginite M200 Nanoquant). The results were presented as mean ± standard deviation.

### 2.10. Statistical Analysis

All data were expressed as average ± standard deviation. Data analysis was performed using Origin Pro 8.5.0. The Student’s *t*-test was used for the statistical analysis among the groups. Statistical significance was considered at *p* < 0.05.

## 3. Results and Discussion

### 3.1. Chemical and Physical Characterization of Star-Shaped PCL and PCL-PEG

**Figure 3:**
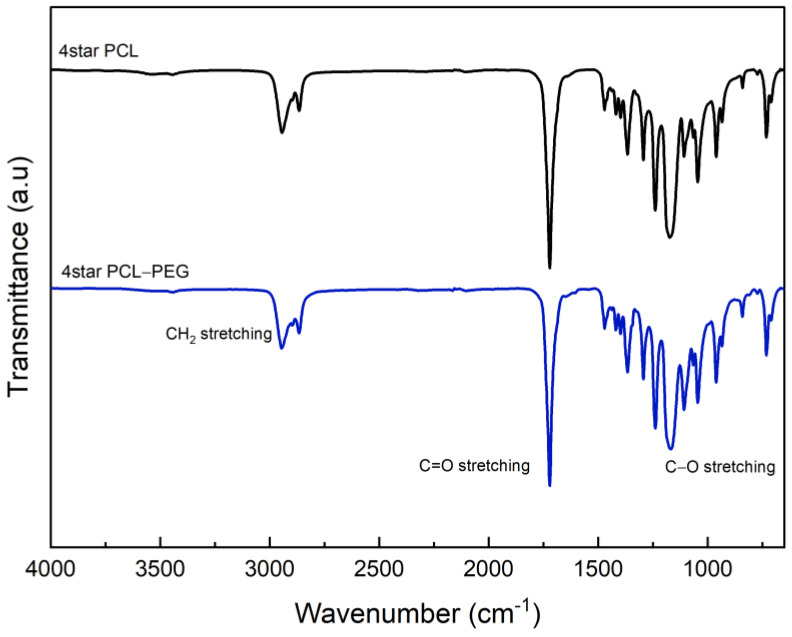
FTIR spectra of 4Star PCL and 4Star PCL-PEG.

FTIR analyses of 4Star-PCL and 6Star-PCL were reported in Figure 3. The spectra confirmed the mechanism of the ring-opening polymerization (ROP) of ɛ-caprolactone via the appearance of the C=O stretching at around 1720 cm^−1^. In both cases, the C-O stretched bands appeared around 1172 cm^−1^. The C-H band of the methyl group of the PCL was detected from 2943 to 2865 cm^−1^ in the case of 4Star PCL and from 2944 cm^−1^ to 2865 cm^−1^ in the case of 6Star PCL. The presence of a PCL backbone band confirmed the ROP of cyclic ɛ-caprolactone. The presence of a weak hydroxyl band at ~3500 cm^−1^ is probably due to the low content of the -OH in the compounds [34,35].

**Figure 4:**
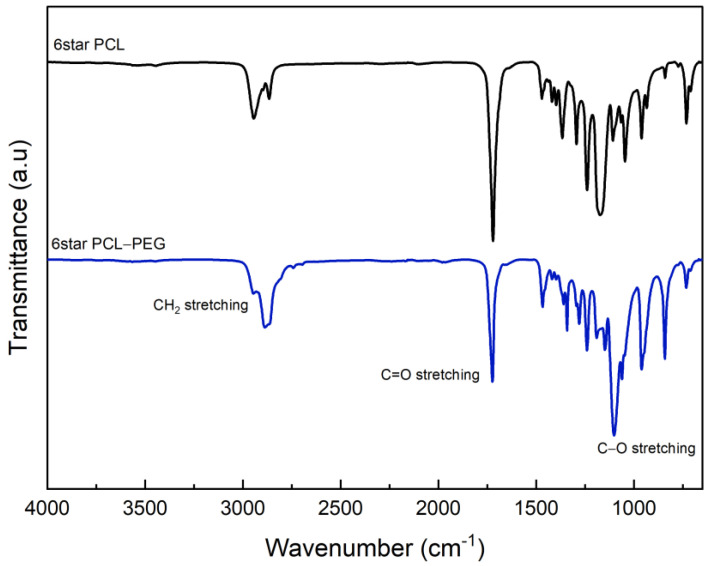
FTIR spectra of 6Star PCL and 6Star PCL-PEG.

FTIR spectra of 4Star PCL-PEG and 6Star PCL-PEG were reported in Figure 4. No relevant differences were recognized due to the presence of similar functional groups. In particular, both spectra do not show the presence of -OH bands. The C=O band stretching of 4Star PCL-PEG appeared at 1720 cm^−1^, while the bands for C-H stretching appeared at 1294 and 2865 cm^−1^. The band at 1167 cm^−1^ was due to C-O-C stretching vibrations of the repeated -OCH_2_CH_2_ units, consistent with the addition of PEG ether units. The band at 1293 cm^−1^ was attributed to the -COO- stretching vibrations. The C=O stretching band of 6Star PCL-PEG appeared slightly shifted at 1723 cm^−1^, and the C-H stretching was shown at 2887 cm^−1^. The C-O-C and -COO- functional groups appeared at 1279 and 1101 cm^−1^, respectively. Similar findings were reported by other researchers [34,36].

The ^1^H NMR spectra of 4Star-PCL and 6Star-PCL were reported in Figure S1. The chemical shifts at 4.05 ppm (t, -CH_2_-O-) and 3.6 ppm (t, -CH_2_-OH) denote the methylene protons of ethers from the backbone and the terminal end group for the polymer arm, respectively. The multiplets at chemical shifts from 1.3 to 2.4 ppm [δ1.6, δ1.3 (m, 2H, 2-CH_2_-),(t, 2H, -CH_2_-)] are referred to PCL backbone, confirming ROP of ɛ-caprolactone monomer. The polymerization of each arm from the core is assumed to be the same since the active group of each arm is the hydroxyl group that initiates the ROP of caprolactone [34,36]. NMR data clearly indicated that the ROP occurred on each arm of both macroinitiators, which then polymerized to form the star-shaped homopolymers of PCL.

^1^H NMR spectra for 4Star PCL-PEG and 6Star PCL-PEG polymers were reported in Figures S2 and S3. The chemical shifts from 1.3 to 2.4 ppm [δ1.3, δ1.6 (m, 2H, 2 -CH_2_-); δ2.3 (t, 2H, -CH_2_-)] denotes that the PCL backbone is attached to the central core. A triplet at 2.65 ppm (t, -COCH_2_CH_2_CO-) denotes the methylene group linker. This peak is slightly shifted to a downfield due to the de-shielding effect after PCL backbone attachment. A singlet at 3.4 ppm (m, -OCH_3_) is associated with the methoxy group for the end terminal in mPEG, while chemical shifts from 3.5 to 3.7 ppm (m, -OCH_2_CH_2_OCH_3_) are referred to as poly (ethylene glycol) backbone. Meanwhile, a triplet at 4.0 ppm (t, -CH_2_O-) is associated with the methylene ester on the PCL backbone [31]. The 1H NMR spectra of 4Star and 6Star PCL-PEG show additional peaks attributed to the PEG structure, which signify the successful conjugation of PCL and PEG via esterification to form the block star co-polymers.

**Figure 5:**
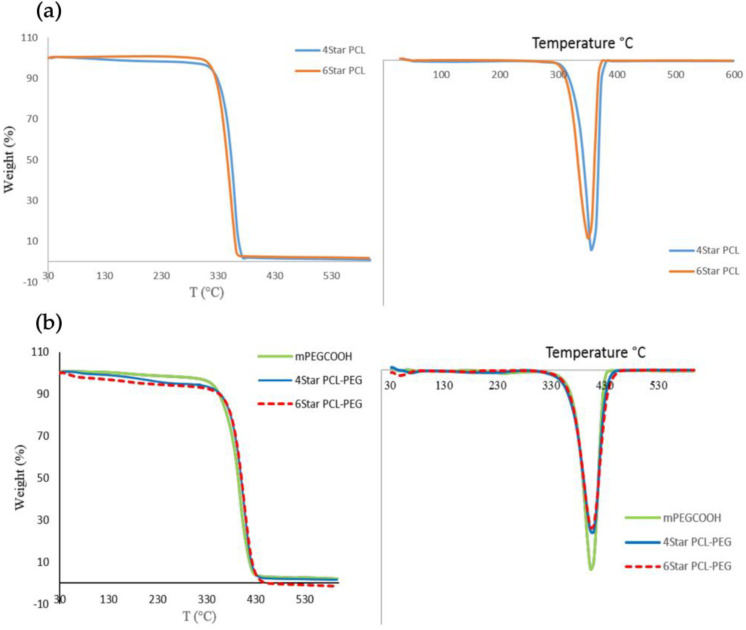
TGA and DTG thermograms: (**a**) 4Star PCL and 6Star PCL; (**b**) 4Star PCL-PEG and 6Star PCL-PEG.

The thermal properties of the synthesized four- and six-arm star-shaped block co-polymer were examined using thermogravimetric analysis (TGA/DTG) and differential scanning calorimetry (DSC). The initial decomposition for both 4Star PCL and 6Star PCL is about 310 °C (Figure 5).

Even though both PCL star homopolymers are approximately the same in terms of molecular weight, the length for each six- and four-arm star-shaped polymer is different, depending on its degree of polymerization. The star polymers PCL-PEG showed high thermal stability based on an initial decomposition temperature of 340 °C. The addition of mPEG-COOH to both star PCL increased the thermal stability of the polymer compared to the homopolymer itself. These thermal changes were expected since the addition of PCL increased the decomposition temperature of the co-polymers due to the high thermal stability of PEG [37]. Star co-polymers have stable terminal group chains (-OCH_3_), leading to higher thermal stability compared to star PCL homopolymers due to hydroxyl terminal end groups (-OH), which are prone to rapid thermal degradation. The similar degradation rates in both star PCL homopolymers and star PCL-PEG are due to the same molar ratio of both PCL and PEG, even though they have different architectures. The results showed that the polymer architecture did not significantly affect the thermal degradation of the polymers. The observed differences in thermal stabilities of the synthesized polymers are consistent with their structures.

Thermal analysis using DSC was used to gain insight into the self-organization and the crystallization behavior of the star-shaped co-polymers in bulk. The melt crystallization of these co-polymers is rather complex since both PCL and PEG have close melting temperatures that lead to the occurrence of coincident crystallization of both components, or the crystallization of one block may affect the crystallization of the second block [38,39,40]. All samples showed no significant increase or decrease in crystalline melting point (Table 1) of each polymer and block co-polymer.

**Figure 6:**
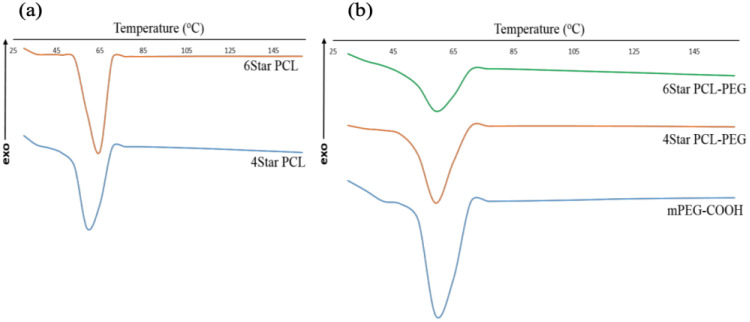
DSC thermograms of (**a**) star PCL and (**b**) star PCL-PEG.

Both 4Star PCL and 6Star PCL were observed to possess crystalline melting points within the melting range of PCL (Figure 6). These block co-polymers are known to phase separately at certain PCL: PEG ratios and PCL lengths. Generally, where there is a high proportion of PCL, it blocks the crystallization of PEG. The T_m_ values of these co-polymers are very close to each other (59–60 °C), thus showing that the architecture does not significantly affect the thermal behavior of PCL-PEG star-shaped block co-polymers. Based on the DSC value, both block co-polymers gave slightly lower T_m_ compared to mPEG-COOH. Such a finding may be related to the higher molecular weight of PCL blocks, which crystallized first and consequently disturbed the organization of the PEG segment that altered the T_m_ value of PEG. This also shows that the T_m_ of PCL also were not affected by the increased PEG content since 6Star PCL-PEG has a higher number of PEG block compared to 4Star PCL-PEG. The T_m_ value of PCL in block co-polymers was almost identical to its star-shaped homopolymers, suggesting that PCL crystallization is barely affected by the presence of the PEG segment. Since there was no significant observable mPEG melting peaks in the co-polymer samples, it can be concluded that both star block co-polymers consisted of a semi-crystalline PCL phase, while PEG dispersed within the part of the semi-crystalline PCL phase [38].

### 3.2. Ciprofloxacin Loaded Star Polymers PCL-PEG

Two formulations were prepared by loading ciprofloxacin in 4Star PCL-PEG (4SF) and 6Star PCL-PEG (6SF), respectively. The main challenge in preparing hydrogel formulation containing PCL is the hydrophobicity of PCL, and the co-polymerization of PCL with PEG is to increase the solubility of the star polymer in water to be incorporated into the formulation. TFE was chosen as the solvent to dissolve the star polymers and ciprofloxacin before mixing it with a water base carbomer. The ratio of the ciprofloxacin and TFE used is very crucial to make sure that the hydrogel formulation is in a homogenous form. After the addition of water to the mixture of star polymer, drugs, and carbomer, an opaque hydrogel formed with a smooth and homogenous appearance without any agglomeration. However, formulations that contain star-shaped homopolymer PCL, either four or six arms, did not form a homogeneous formulation as the polymer tends to agglomerate in the hydrogel. The introduction of hydrophilic polyether blocks, PEG, into PCL chains is a means to enhance the hydrophilicity of the star polymers compared with the homopolymer to form a homogenous mixture in the formulation. The hydrophobic blocks associate to form the inner region, whereas the position of the hydrophilic segments between the inner segment and the external aqueous medium led to an increase in hydrophilicity. Hence, the hydrophobic segment is stabilized via the hydrophilic outer segment, which serves as an interface between the bulk aqueous phase and the hydrophobic domain [40]. The amount of TEA added to each formulation was based on the initial pH of the formulation until it reaches a pH in the range between 7.2 and 7.4 ± 0.03. The viscosity of each formulation was between 9100 and 9400 centiPoise. The observation of the hydrogel formulations for three weeks shows no physical changes for all formulations, such as color and homogeneity.

### 3.3. Encapsulation Efficiency

All hydrogels have shown high drug entrapment efficiency with more than 98% encapsulation (Table 2), indicating high entrapment efficiency. Both hydrogels showed high drug-loading capacity since the star polymers serve as a larger reservoir for encapsulating the drug. The hydrophobic PCL arms in the star polymers likely led to the formation of a larger polymeric star inner region that readily encapsulates the drug. The hydrophobicity of the drug also affects the drug entrapment within the star polymer, which is consistent with the previous studies [41,42]. The property leads to hydrophobic–hydrophobic interactions between ciprofloxacin and the PCL segment in the star-shaped polymer that increase the drug loading of the formulation. The number of arms of a star polymer, which is related to the polymer architecture, did not significantly contribute to the changes in the drug entrapment efficiency, as there is no significant difference observed in both hydrogels. It is, therefore, concluded that the encapsulation capacity is based on the hydrophobic part of the star-shaped polymer and the hydrophobicity of the drug itself [43].

### 3.4. Drug Release

**Figure 7:**
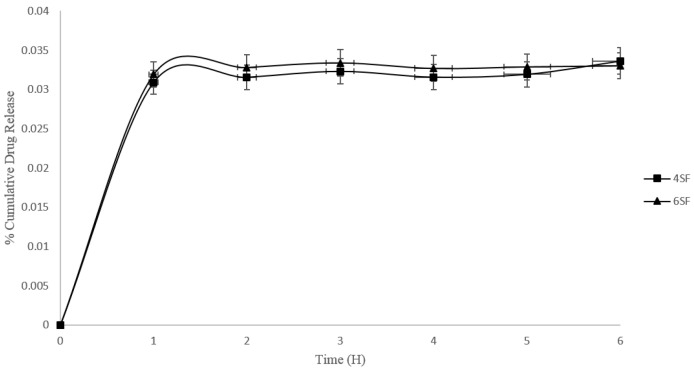
Cumulative drug release vs. time for 4SF and 6SF formulations.

The release of drugs is typically influenced by initial diffusion, followed by a combination of diffusion and degradation mechanisms [44]. Both star polymer hydrogels also showed sustained release behavior (Figure 7). The drug release begins at the sphere surface, followed by the release from the inner layers of the sphere [44], and exhibits an initial burst release in both formulations. It showed the dispersion of ciprofloxacin towards the center of the star polymer, which results in a more constant release rate. The hydrophobic drug tends to be attached to the hydrophobic segment in the star polymer rather than the hydrophilic outer segment. The sustained release pattern of the formulations is related to the presence of PCL in the star-shaped polymer and the drug hydrophobicity, which is related to the hydrophobic–hydrophobic conjugation between the PCL segments. The PCL degradation has been reported to be very slow in an aqueous medium because of its semi-crystallinity and hydrophobicity. Thus, their drug-releasing behaviors would not be seriously affected by the degradation of PCL blocks. However, water can penetrate the amorphous regions of the polymer matrix, facilitating the slow release of ciprofloxacin via diffusion due to the ciprofloxacin having low solubility in the system [45]. The PCL properties result in a longer duration of ciprofloxacin intake that leads to a sustained release behavior.

Even though the 4SF and 6SF samples have similar release behaviors, the 6SF showed a slightly higher amount of drug released compared to the 4SF. This behavior may be attributed to the 6SF architecture, which has a relatively longer PCL block length and a higher number of PEG compared to 4SF. Mishra et al. [46] suggested that a longer PCL block may restrict the permeation of water molecules across the polymeric matrix, which causes the slow hydration of the gel due to the decreased diffusion coefficient of the drug across the hydrogel matrix, contradicting our 6SF results. Hence, we postulated that the higher amount of drug release in 6SF was due to the higher number of PEG, which increased the water intake within the start polymer and caused the higher diffusion release of ciprofloxacin in the 6SF formulation.

### 3.5. Kinetic Model

The drug-release data of the formulations were examined for the applicability of various mathematical kinetic models to consider the suitable dissolution profiles of the samples [33,47]. The data from the hydrogel were fitted into zero-order, first-order, Higuchi, and Korsmeyer–Peppas mathematical models to observe the drug-release mechanism referred to in Figures S4 and S5. The coefficient R^2^, from Table 3, obtained from the graphs, shows the best linearity in both 4SF and 6SF in the Korsmeyer–Peppas model. It can be concluded that both formulations are best fitted in the Korsmeyer–Peppas release model compared to the other mathematical modeling.

The “n” value (Table 4) for the 4SF and 6SF formulations determined from the slope of the plot were 0.0054 and 0.0159, respectively. When the “n” value is exceptionally low, it suggests that the drug release is predominantly governed by a super case II transport mechanism. This type of mechanism typically indicates a release behavior that deviates significantly from Fickian diffusion, where the drug-release rate is not solely dependent on the concentration gradient but may involve additional factors like erosion, swelling, or relaxation of the matrix material. Regardless of the solubility of the antibiotic, the release of drugs from the hydrogel was most likely regulated via a diffusion mechanism [48,49].

Fick diffusion suggested that the hydrogel network was relaxed after the dissolution medium penetrated the hydrogel matrix. Then, the drug model was carried out using the medium that entered the matrix through the pores of the hydrogel matrix via diffusion [50,51]. This also proved that the slightly higher release rate in the 6SF formulation was due to the higher number of PEG that would increase the water uptake and hence increase the release rate compared to 4SF. Based on the data model, the probable ciprofloxacin released from 4SF and 6SF formulations was governed via diffusion. The degradation release factor of the polymer is not applicable since PCL has a low degradation rate and may not significantly affect the release rate of the drug model. The Korsmeyer–Peppas model was developed to specifically model the release of a drug molecule from a polymeric matrix, such as a hydrogel [49]. Even though the R^2^ values obtained from the mathematical modeling are not significantly closer to 1, the data serve as a preliminary study to obtain novel insight into the release kinetics of the drug model from the formulations. This data can be used for the further optimization of the formulation, especially the drug-loading and drug-release capacity, to ensure that it can be used effectively toward targeted application.

### 3.6. Antimicrobial Activity

The ability of the formulations to inhibit four bacterial strains (*E. coli*, *P. aeruginosa*, *E. faecalis*, and *S. pyogenes*) was performed via the zone of inhibition testing. All formulations containing ciprofloxacin showed activity against the selected Gram-positive and Gram-negative bacteria (Table 5). In the first 3 h of incubation, all formulations did not show any inhibition, particularly for formulations containing ciprofloxacin, indicating no initial burst of the drug from the cargo. At 6 h, all formulations started to show bacterial inhibition for all microorganisms except for STN4 and STN6 for *P. aeruginosa* and *E. faecalis*. After 12 h of incubation, the zone of inhibitions for formulations with the antibiotic is doubled compared to 6 h of incubation. The results indicate the controlled drug release of the formulations towards ciprofloxacin. Meanwhile, STN4 and STN6 showed only a small increase in the inhibition zone of *E. coli* and *S. pyogenes* at 12 h. The highest bacterial inhibition for both formulations with ciprofloxacin after 24 h of incubation is *E. coli*.

4SF and 6SF formulations have approximately similar inhibitions, suggesting that both formulations contain the same amount of drug encapsulated as calculated in the formulation encapsulation efficiency. After 24 h of incubation, there was a slight increase in bacterial inhibition for 4SF and 6SF. However, there is no change in inhibition observed for STN4 and STN6 from 6 h to 24 h. STN4 and STN6 also showed bacterial inhibition for *E. coli* and *S. pyogenes*, even though they did not contain ciprofloxacin. This is due to the presence of parabens as preservatives in the formulations. However, parabens show weak inhibitory effects and are insignificant against *E. coli* and *S. pyogenes* [52]. Methyl and propyl parabens are also more effective against fungi compared to bacteria [53]. *P. aeruginosa* and *E. faecalis* were reported to be resistant to parabens as well [54], and the absence of inhibitions for these two microorganisms in both STN4 and STN6 also demonstrates that the small inhibition zone observed in *E. coli* and *S. pyogenes* was due to the parabens. Hydrogels containing Cipro show good antibacterial activity towards all bacteria tested and are in agreement with the study by Asghar et al. [55].

### 3.7. Cell Biocompatibility Studies

**Figure 8:**
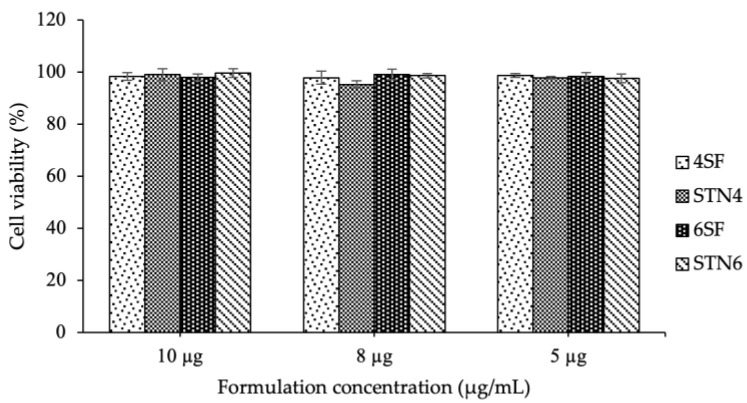
Cytotoxicity tests of HGF-1 cells on media with different formulations, after 24 h exposure.

The in vitro biocompatibility was analyzed using HGF-1 in the culture media containing different hydrogel formulations investigated in this study. The cytotoxicity test was performed to investigate the effects of ciprofloxacin as an antibiotic in different concentrations. One group of cell cultures was treated with DMEM with no added formulations, to which the cell number of other types of DMEM containing the formulations was compared. Figure 8 shows the number of living cells measured 24 h after the cell cultures were exposed to the formulation’s environment compared to the control groups. After the treatment of HGF-1 cell cultures with the hydrogels, the cells did not show any significant difference in the four hydrogels compared to the control cells. As seen in Figure S6, the morphology of cell lines after treatment of 10 µg/mL of formulation and control cells showed that all formulations have good biocompatibility towards normal cells. The hydrogel concentrations gave high cell viability, which is more than 96%. The cellular activity on the hydrogels was within the same range as the cell cultures without treatment. These data suggest that all hydrogels containing ciprofloxacin have good biocompatibility and non-toxic response with respect to the controls [56].

## 4. Conclusions

In summary, the amphiphilic star polymers of four- and six-arm PCL-PEG with different architectures were successfully synthesized and fully characterized via ^1^H-NMR, FTIR, TGA, and DSC. Ciprofloxacin was loaded into the star polymers PCL-PEG in the hydrogel form. The presence of -PEG in the outer block of the star polymers promotes blending. Meanwhile, the hydrophobic PCL tends to agglomerate and form non-homogeneous hydrogels, with effects on drug-loading and drug-release behavior. It is known that the combination of two polymers can produce gels that have different degrees of rigidity as well as stability. In this study, both hydrogels have shown high loading efficiency (more than 90%), in six-arm star polymers hydrogels slightly higher than the four-arms star polymer ones. Sustained drug release, reported for star polymer-based hydrogels, is well fit via the kinetics model following the Korsmeyer–Peppas release profile. An antibacterial study of the hydrogels confirmed good antimicrobial activity due to the action of ciprofloxacin and its sustained release from the hydrogels. The cytotoxicity tests showed a low cytotoxic response of the hydrogels suitable for biomedical applications. Hence, multiple arm star polymers can be considered promising for the development of novel carriers for oral delivery applications.

## Figures and Tables

**Table 1:** Summary of characteristic temperatures of star polymers.

Sample	T_m_ ^a^ (°C)	T_d-max_ ^b^ (°C)
4Star PCL	60	356.6
6Star PCL	56	327.5
4Star PCL-PEG	59	409.2
4Star PCL	60	409.1

^a^ T_m_ = melting transition temperature obtained from DSC thermograms. ^b^ T_d-max_ = temperatures of maximum decomposition obtained from DTG thermograms.

**Table 2:** Drug entrapment efficiency of hydrogel formulations.

Formulations	% Drug Entrapment
4SF	99.20 ± 0.01
6SF	99.25 ± 0.04

**Table 3:** Coefficient R^2^ of suggested modeling.

Formulations	Coefficient, R^2^			
	Zero Order	First Order	Higuchi	Korsmeyer–Peppas
4SF	0.429	0.621	0.429	0.651
6SF	0.394	0.269	0.394	0.680

**Table 4:** Diffusion exponent value n based on Korsmeyer–Peppas.

Formulations	Coefficient, R^2^	n
4SF	0.651	0.005
6SF	0.680	0.0159

**Table 5:** Inhibition zone of all hydrogels.

	Microorganisms															
	*Escherichia coli*				*Pseudomonas aeruginosa*				*Enterococcus faecalis*				*Streptococcus pyogenes*			
	Inhibition Zone (mm)				Inhibition Zone (mm)				Inhibition Zone (mm)				Inhibition Zone (mm)			
Time (Hour)	3	6	12	24	3	6	12	24	3	6	12	24	3	6	12	24
4SF	X	16.3	36.3	44.3	X	12.3	28.3	30.3	X	14.3	29.0	31.0	X	16.3	33.3	37.3
6SF	X	16.7	35.3	43.3	X	12.7	28.7	31.0	X	13.7	28.0	31.0	X	15.7	34.7	38.3
STN4	X	11.3	14.6	14.6	X	X	X	X	X	X	X	X	X	9.7	12.0	12.0
STN6	X	12.0	16.3	16.3	X	X	X	X	X	X	X	X	X	10.9	12.3	12.3

X—no inhibition. 4SF—Hydrogels contain four-arm star-shaped PCL-PEG and ciprofloxacin. 6SF—Hydrogels contain six-arm star-shaped PCL-PEG and ciprofloxacin. STN4—Hydrogels contain four-arm star-shaped PCL-PEG. STN6—Hydrogels contain six-arm star-shaped PCL-PEG.

## Data Availability

Not applicable.

## Supplementary Materials

The following supporting information can be downloaded at: https://www.mdpi.com/article/10.3390/mi14071382/s1, Figure S1: Calibration curve of ciprofloxacin; Figure S2: ^1^H NMR spectra of (a) 4Star PCL and (b) 6Star PCL in CDCl_3_; Figure S3: ^1^H NMR spectra of (a) 4Star PCL-PEG and (b) 6Star PCL-PEG in CDCl_3_; Figure S4: Graph of mathematical kinetic model for 4SF; Figure S5: Graph of mathematical kinetic model for 6SF; Figure S6: Morphology of cell lines (a) 10 µg/mL SF4, (b) 10 µg/mL STN4, (c) 10 µg/mL SF6, (d) 10 µg/mL STN6 and (e) control cell after 24 h of treatment.
