# Supplementary material for: Synthesis and Characterization of Ciprofloxacin Loaded Star-Shaped Polycaprolactone–Polyethylene Glycol Hydrogels for Oral Delivery
Source: Micromachines (Basel). 2023 Jul 6;14(7):1382. doi: 10.3390/mi14071382 (PMC10383659; doi:10.3390/mi14071382)
Supplement: Supplementary file 1 [file micromachines-14-01382-s001.zip › micromachines-2417821-supplementary.pdf]

Supplementary Files

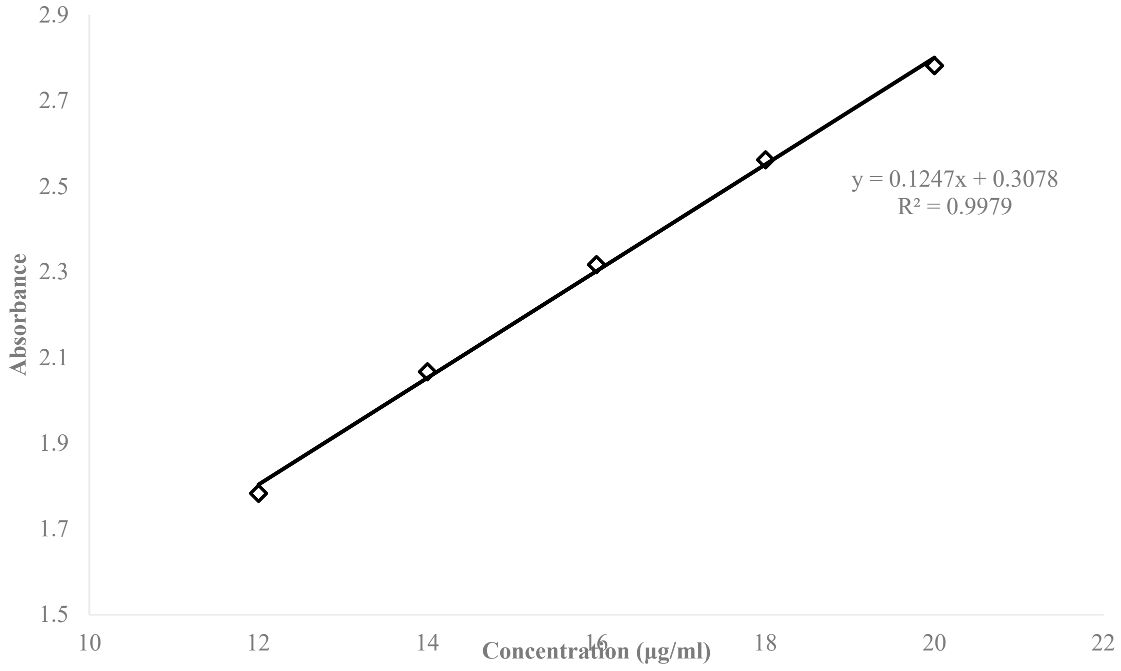

Figure S1 Calibration curve of ciprofloxacin

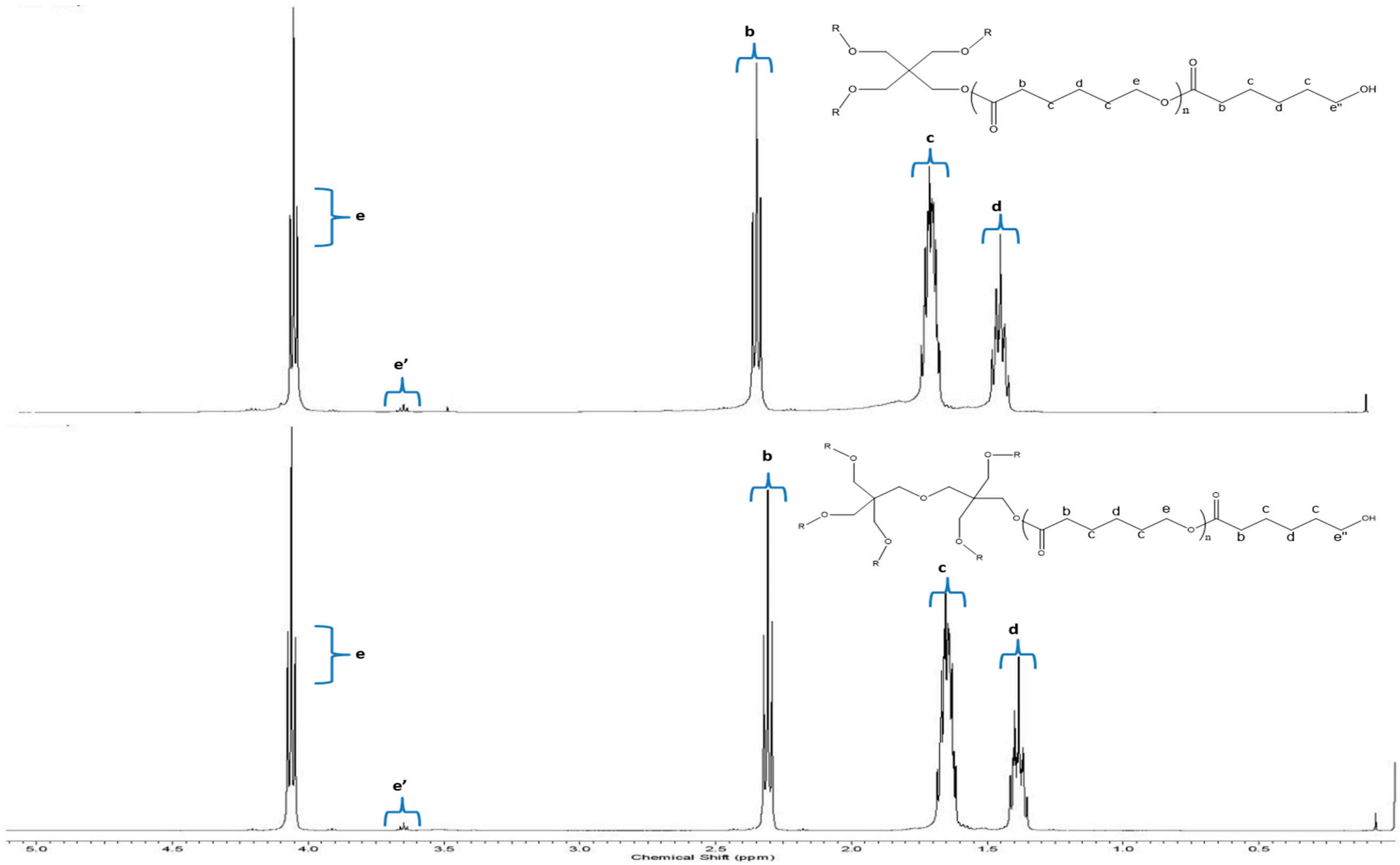

Figure S2: ^{1}H NMR spectra of a) 4Star PCL and b) 6Star PCL in CDCl3

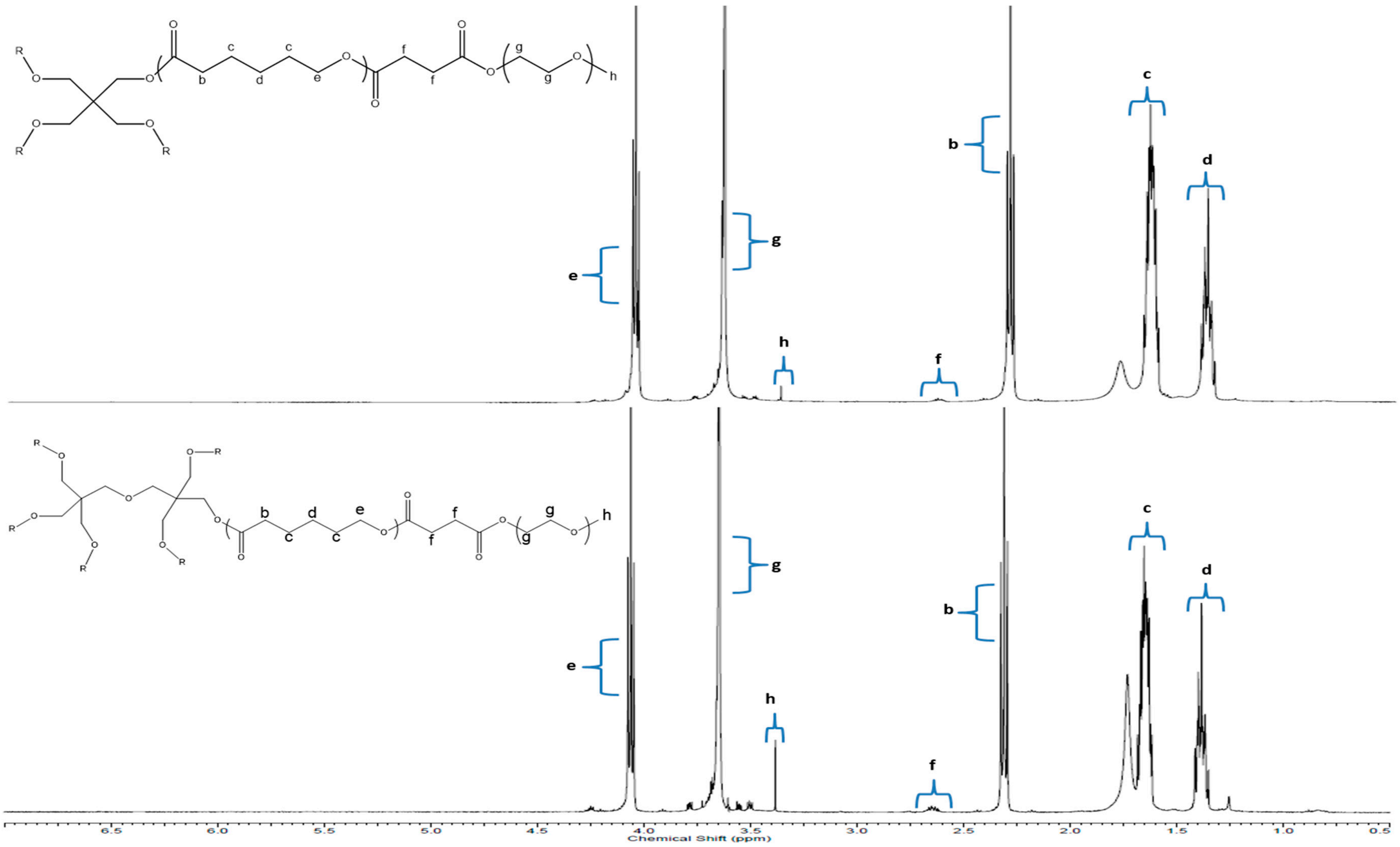

Figure S3: ^{1}H NMR spectra of a) 4Star PCL-PEG and b) 6Star PCL-PEG in CDCl3.

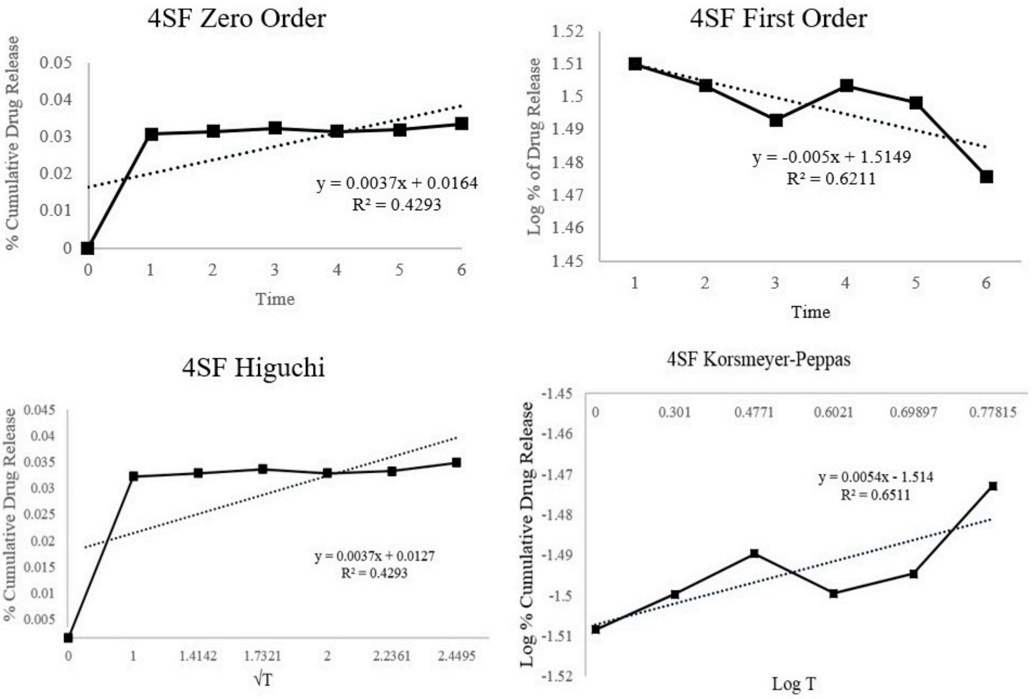

Figure S4: Graph of mathematical kinetic model for 4SF.

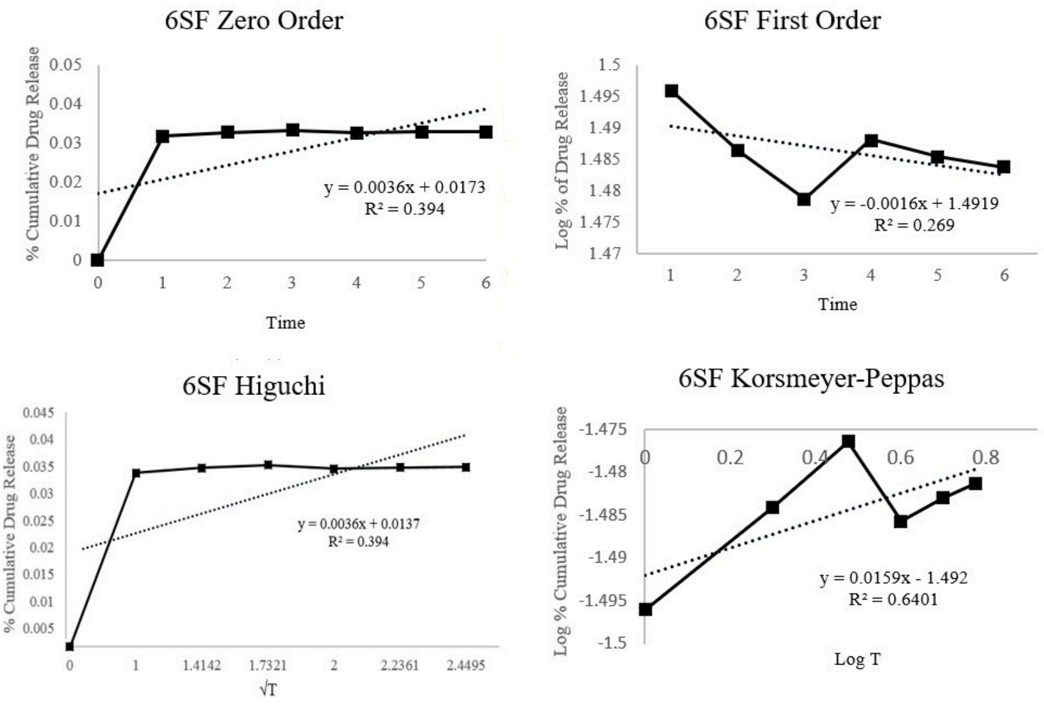

Figure S5: Graph of mathematical kinetic model for 6SF.

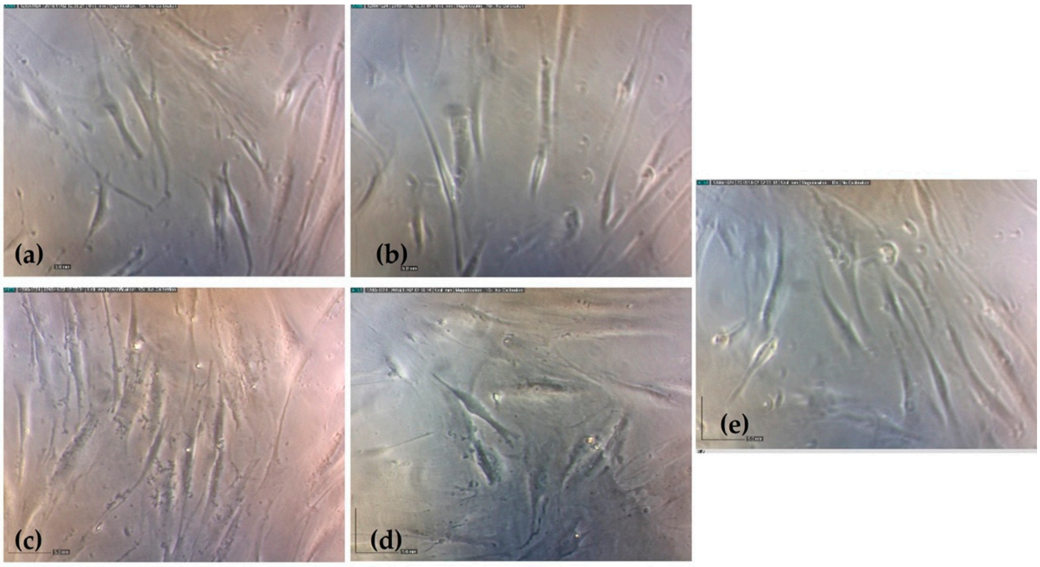

Figure S6**:** Morphology of cell lines a) 10 µg/mL SF4, b) 10 µg/mL STN4, c) 10 µg/mL SF6, d) 10 µg/mL STN6 and e) control cell after 24 hours of treatment.
